# Supplementary material for: Development of Liver Fibrosis Represented by the Fibrosis-4 Index Is a Specific Risk Factor for Tubular Injury in Individuals with Type 2 Diabetes
Source: Biomedicines. 2024 Aug 7;12(8):1789. doi: 10.3390/biomedicines12081789 (PMC11352124; doi:10.3390/biomedicines12081789)
Supplement: Supplementary file 1 [file biomedicines-12-01789-s001.zip › biomedicines-2991773-supplementary.pdf]

```
z tests - Logistic regression
Options:   Large sample z-Test, Demidenko (2007) with var corr
Analysis:  Post hoc: Compute achieved power
Input:          Tail(s)                        =     Two
                Odds ratio                     =     1.857
                Pr(Y=1|X=1) H0                 =     0.5010635
                α err prob                     =     0.0033
                Total sample size              =     885
                R² other X                     =     0.07783
                X distribution                 =     Normal
                X parm μ                       =     1.56
                X parm σ                       =     1.11
Output:         Critical z                     =     2.9383164
                Power (1-β err prob)           =     1.0000000
```

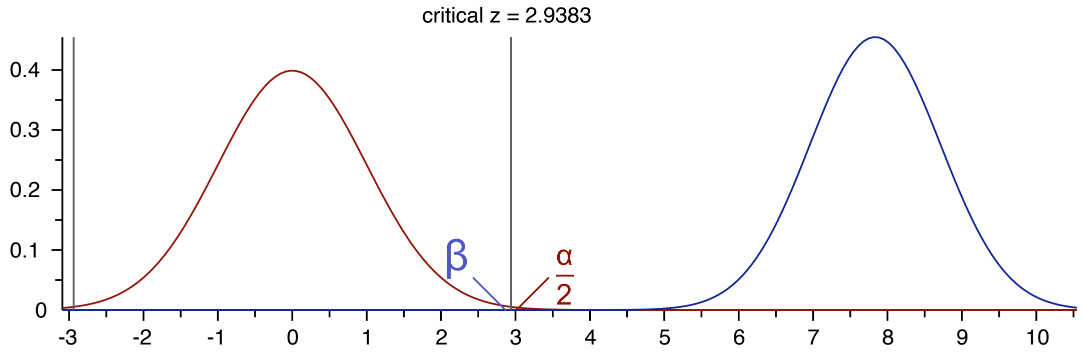

Figure S1 (a). Results of the power analysis of the logistic analysis in glomerular injury specific group

```
z tests - Logistic regression
Options:   Large sample z-Test, Demidenko (2007) with var corr
Analysis:  Post hoc: Compute achieved power
Input:          Tail(s)                        =     Two
                Odds ratio                     =     1.857
                Pr(Y=1|X=1) H0                 =     0.5010635
                α err prob                     =     0.0167
                Total sample size              =     727
                R² other X                     =     0.07783
                X distribution                 =     Normal
                X parm μ                       =     1.55
                X parm σ                       =     0.87
Output:         Critical z                     =     2.9383164
                Power (1-β err prob)           =     0.9990944
```

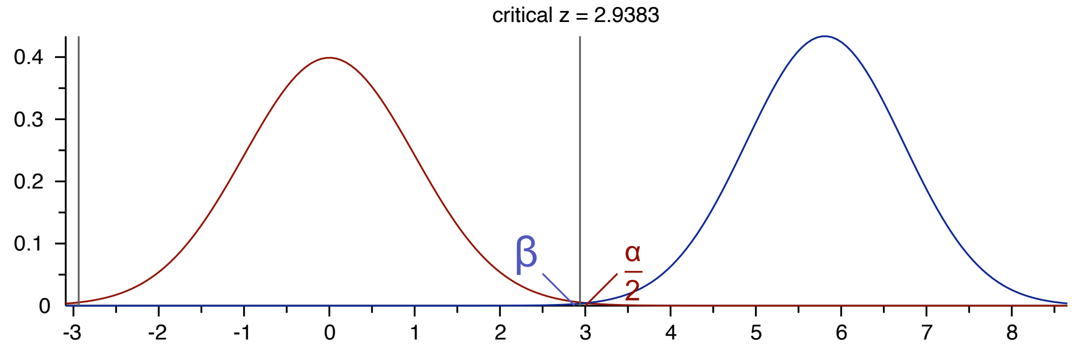

Figure S1 (b). Results of the power analysis of the logistic analysis in tubular injury specific group

```
z tests - Logistic regression
Options:   Large sample z-Test, Demidenko (2007) with var corr
Analysis:  Post hoc: Compute achieved power
Input:          Tail(s)                        =     Two
                Odds ratio                     =     1.857
                Pr(Y=1|X=1) H0                 =     0.5010635
                α err prob                     =     0.0167
                Total sample size              =     905
                R² other X                     =     0.07783
                X distribution                 =     Normal
                X parm μ                       =     1.56
                X parm σ                       =     0.70
Output:         Critical z                     =     2.9383164
                Power (1-β err prob)           =     0.9943522
```

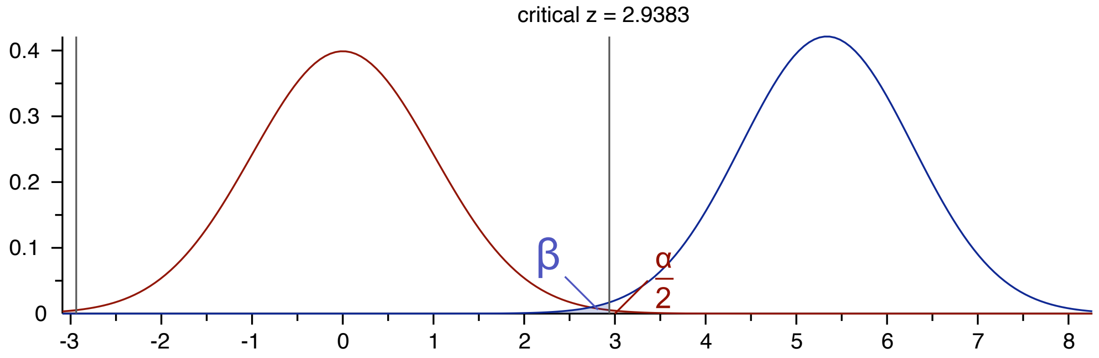

Figure S1 (c). Results of the power analysis of the logistic analysis in dual injury group

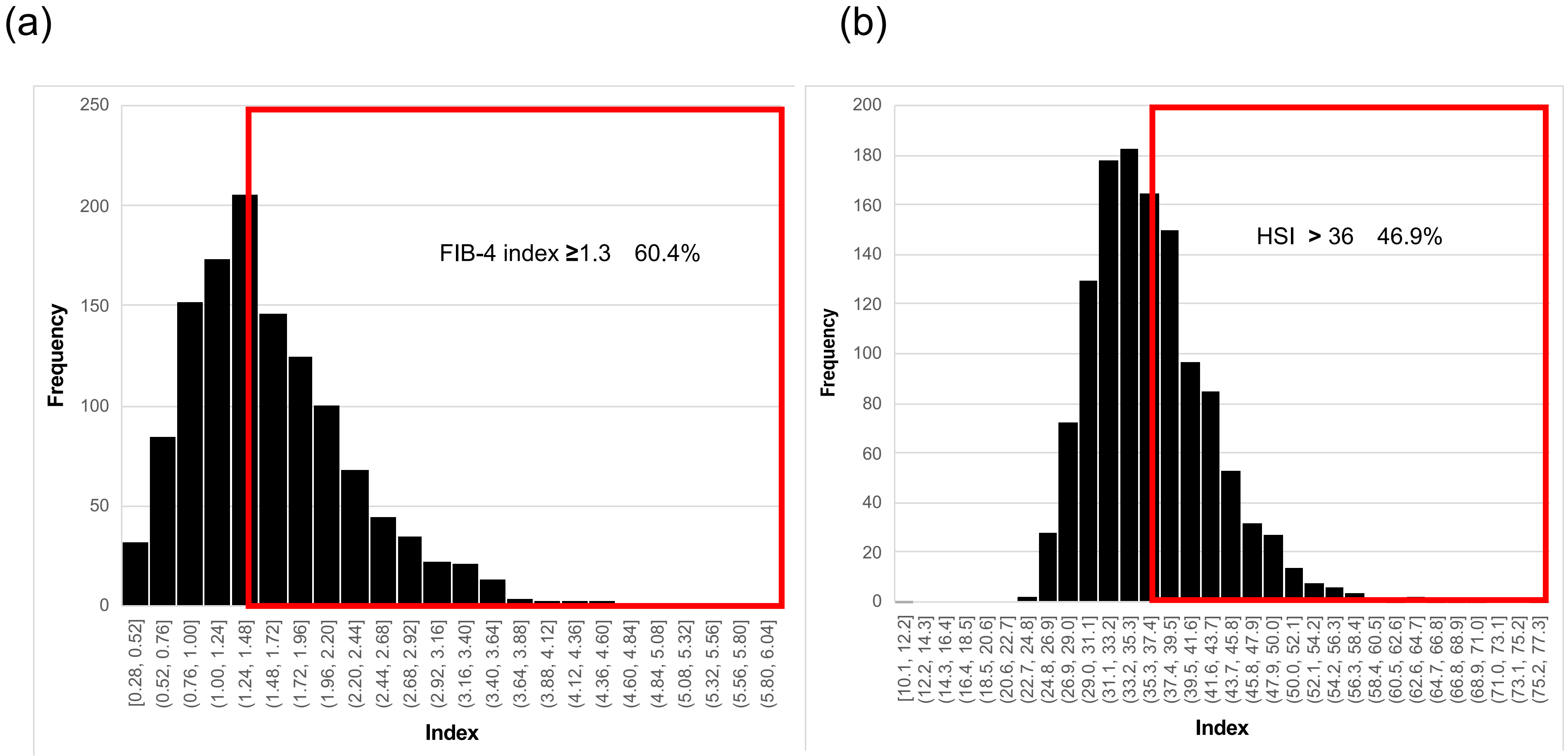

Figure S2. Histogram of the subjects with liver fibrosis or liver steatosis index
(a) FIB-4 index, red-colored frame indicates subjects defined as liver fibrosis
(b) HSI, red-colored frame indicates subjects defined as liver steatosis

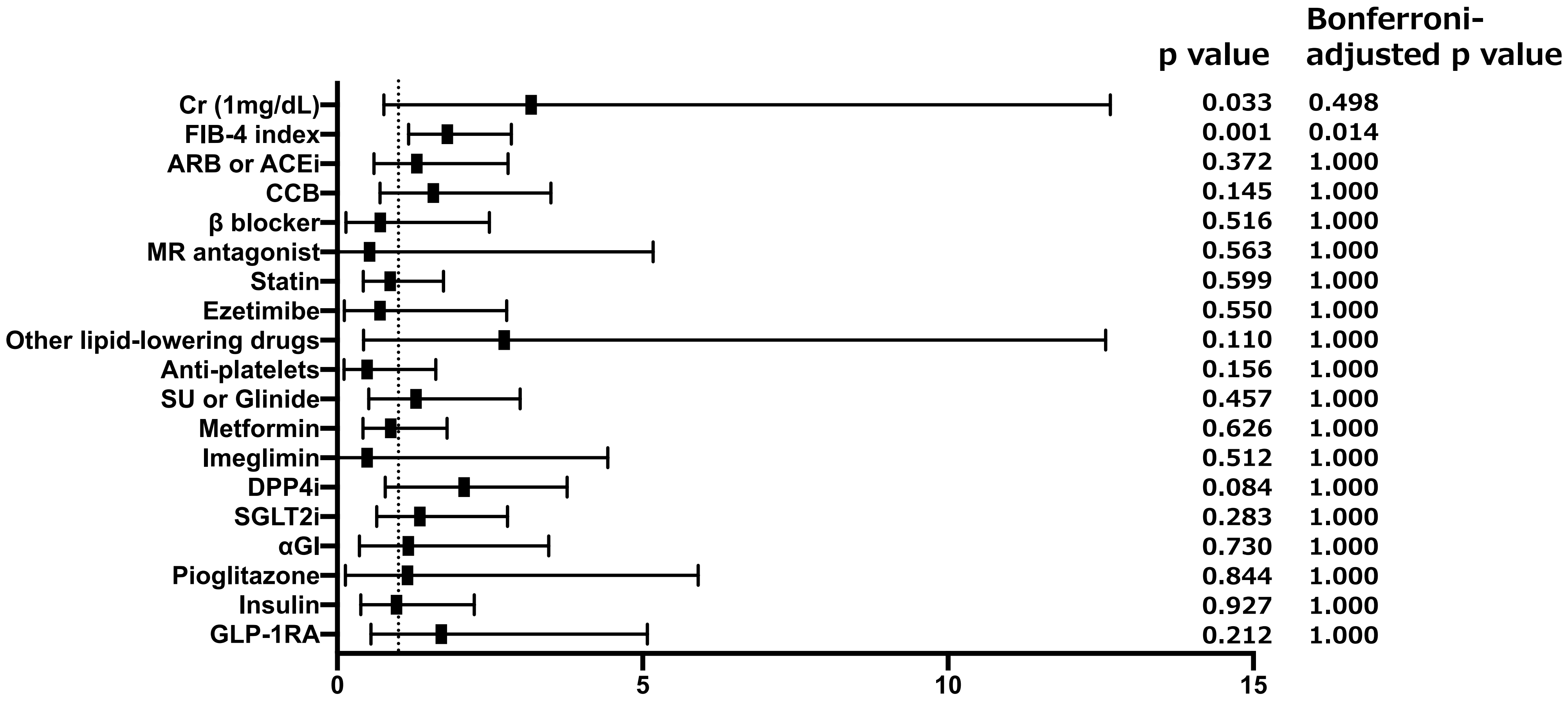

Figure S3. Forest plot of risk factors for prevalence of tubular specific injury defined as uACR < 30mg/gCr and uL-FABPCR ≥ 5μg/gCr after adjusting for identified confounding factors and medications used.

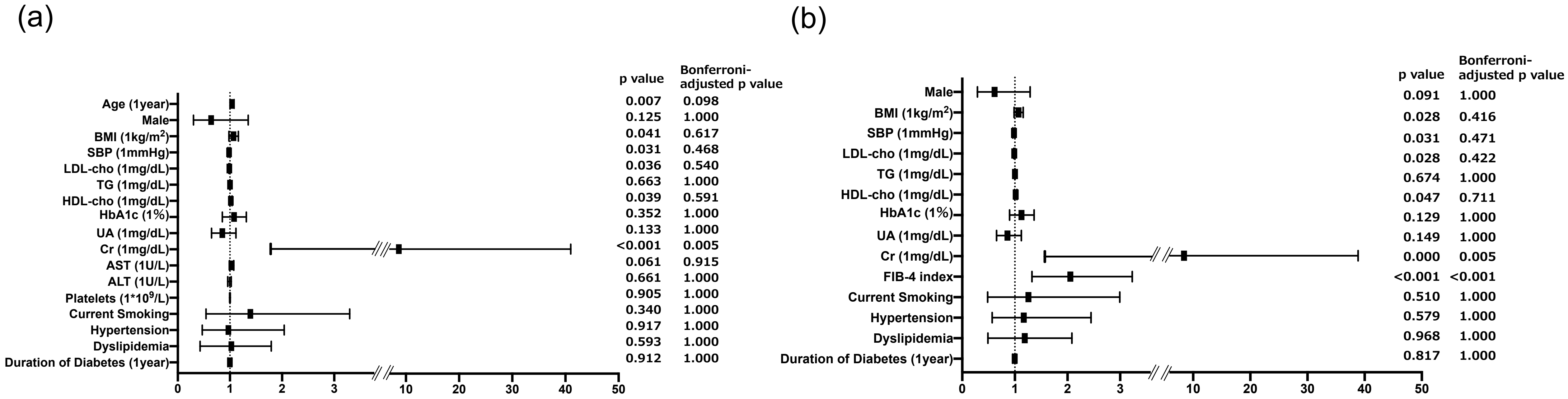

Figure S4. Forest plot of risk factors for prevalence of tubular specific injury defined as uACR < 30mg/gCr and uL-FABPCR ≥ 5μg/gCr

(a) An analysis using AST, ALT, and platelets as covariates instead of the FIB-4 index.

(b) An analysis excluding age from the covariates.

The dashed line in the graph denotes that a portion of the axis is omitted because one of the data points is too large to display properly in the graph.

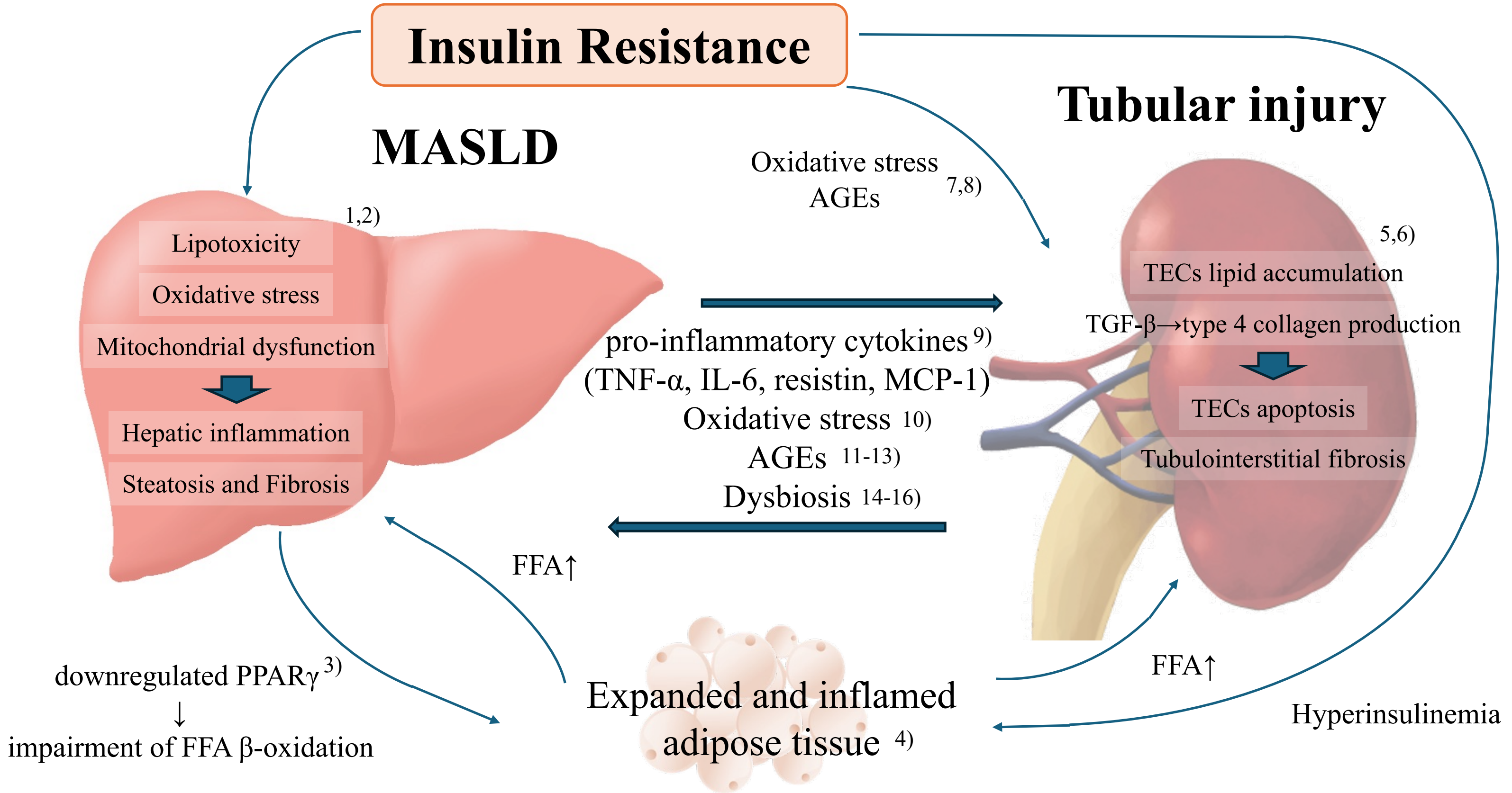

1) Perdomo, C.M et.al., *Nutrients* **2019**, *11*
2) Friedman, S.L et.al., *Nat Med* **2018**, *24*, 908-922
3) Francque, S. et.al., *J Hepatol* **2015**, *63*, 164-173
4) Jia, G et.al., *PLoS One* **2015**, *10*, e0142808
5) Li, X et.al., *Kidney Int* **2024**, *105*, 540-561
6) Chung, K.W. et.al., *J Am Soc Nephrol* **2018**, *29*, 1223-1237
7) Yamagishi, S et.al., *J Biol Chem* **2002**, *277*, 20309-20315
8) Yamagishi, S et.al., *Biomedicines* **2023**, *11*
9) Targher, G et.al., *Semin Thromb Hemost* **2009**, *35*, 277-287
10) Sun, Y et.al., *Cell Death Dis* **2020**, *11*, 914
11) Fernando, D.H.et.al., *Int J Mol Sci* **2019**, *20*
12) Leung, C et.al., *J Hepatol* **2014**, *60*, 832-838
13) Rabbani, N et.al., *Kidney Int* **2018**, *93*, 803-813
14) Sikalidis, A.K et.al., *Biomedicines* **2020**, *8*
15) Chen, P.P. et.al., *Theranostics* **2023**, *13*, 3988-4003
16) Safari, Z et.al., *Cell Mol Life Sci* **2019**, *76*, 1541-1558

Figure S5. Schematic diagram of interaction between MASLD and renal tubular injury in individuals with T2D based on our results and bibliographical considerations.
